# What do we actually know about a common cause of plantar heel pain? A scoping review of heel fat pad syndrome

**DOI:** 10.1186/s13047-022-00568-x

**Published:** 2022-08-16

**Authors:** Alison H. Chang, Steven Zartov Rasmussen, Asger Emil Jensen, Thomas Sørensen, Michael Skovdal Rathleff

**Affiliations:** 1 Department of Health Science and Technology, Faculty of Medicine, Aalborg University, Aalborg, Denmark; 2 Department of Physical Therapy and Human Movement Sciences, Northwestern University Feinberg School of Medicine, 645 N. Michigan Ave. #1100, Chicago, IL 60611 USA; 3 Department of Physio- and Occupational Therapy, Lillebaelt Hospital - University Hospital of Southern Denmark, Vejle, Denmark; 4 Center for General Practice at Aalborg University, Aalborg, Denmark; 5 Department of Physical and Occupational Therapy, Aalborg University Hospital, Aalborg, Denmark

**Keywords:** Heel fat pad, Heel pain, Prevalence, Diagnostic criteria, Intervention, Systematic review

## Abstract

**Background:**

The heel fat pad is an important structure of the foot as it functions as a cushion to absorb shock and distribute plantar force during ambulation. Clinical practice guidelines or decision support platforms emphasize that heel fat pad syndrome (HFPS) is a distinct pathology contributing to plantar heel pain. We aimed to identify and synthesize the prevalence, etiology and diagnostic criteria, and conservative management of HFPS.

**Methods:**

A comprehensive search was conducted in May 2021 and updated in April 2022, using MEDLINE, Scopus, Cinahl, EMBASE, Cochrane Library, SPORTDiscus, and PEDro and ClinicalTrials.gov and the World Health Organization's International Clinical Trials Registry Platform (ICTRP) for pertinent registrations. We included all study types and designs describing the prevalence; etiology and diagnostic criteria; and non-pharmacological, non-surgical interventions for HFPS.

**Results:**

We found a small body of original research for HFPS (*n* = 7). Many excluded full-text articles were expert-opinion articles or studies of heel fat pad in participants with plantar fasciitis/fasciopathy or unspecified heel pain. HFPS may be the second leading cause of plantar heel pain, based on two studies. A number of differentiating pain characteristics and behaviors may aid in diagnosing HFPS vs. plantar fasciopathy. Thinning heel fat pad confirmed by ultrasonography may provide imaging corroboration. Randomized controlled trials assessing the efficacy of viscoelastic heel cups or arch taping for managing HFPS do not exist.

**Conclusions:**

The research literature for HFPS is sparse and sometimes lacking scientific rigor. We have identified a substantial knowledge gap for this condition, frequent inattention to distinguishing HFPS from plantar fasciopathy when describing plantar heel pain, and an absence of robust clinical trials to support the commonly recommended conservative management of HFPS.

## Introduction

Plantar heel pain is the most common musculoskeletal complaint in the foot and accounts for more than 1 million outpatient visits annually [1]. Estimated population prevalence ranged from 3.6% to 7.3% in adults aged 18 and older [2, 3] and 9.6% to 11.1% in middle-aged and older adults [4, 5]. Plantar heel pain is an umbrella term covering different diagnoses, such as plantar fasciopathy/fasciitis, heel fat pad syndrome (HFPS), nerve irritation, calcaneal stress fracture, and lumbar radiculopathy [6–8]. Prior investigations have focused on plantar fasciopathy, although HFPS seems to be a common cause of plantar heel pain [4, 9]. A systematic overview on evidence-based knowledge of HFPS is greatly needed to fill the gaps and inform clinical practice and future research directions.

The heel fat pad is an important structure of the foot as it functions as a cushion to absorb shock and distribute plantar force during ambulation. Clinical commentaries and expert opinions have suggested that aging, injury, repetitive or prolonged overloading (e.g., endurance runners), overweight, improper footwear, steroid injection, and comorbidities (e.g., diabetes, rheumatic diseases) may negatively impact the structure and function of the heel fat pad [8, 10]. These proposed risk factors and structural changes may be part of the etiology of HFPS, however it is unclear if this information is based on high-quality evidence. Unlike plantar fasciopathy, the diagnostic criteria for HFPS have not been well established. Universally agreed diagnostic parameters for HFPS is critical for standardized inclusion and exclusion criteria in epidemiological studies and clinical trials.

Rest, activity modification, icing, analgesics, low-dye arch taping, and viscoelastic heel cups have been recommended to conservatively manage HFPS. Clinical practice guidelines [7] or decision support platforms (e.g., UpToDate [11]) for plantar heel pain emphasize the importance of differentiating HFPS from plantar fasciopathy and propose conservative treatment options despite scant supporting research. Currently, there is limited evidence on the effectiveness of these recommended approaches. Nor have these studies been systematically examined.

HFPS is a distinct pathology contributing to plantar heel pain. Its etiology and diagnostic criteria are poorly understood, and the effectiveness of conservative treatment strategies has not been rigorously examined. Our initial, cursory survey of the literature revealed a lack of systematic overview and limited high-quality evidence in this topic. We conducted a scoping review to systematically identify and synthesize evidence on the prevalence, etiology and diagnostic criteria, and non-pharmacological, non-surgical management of HFPS. These findings will inform future research directions and study designs.

## Methods

### Protocol and registration

We followed the Preferred Reporting Items for Systematic Reviews and Meta-analyses extension for Scoping Reviews checklist (PRISMA-ScR) [12]. This checklist contains 20 essential items plus 2 optional items. The final study protocol was prospectively registered on the Open Science Framework (https://osf.io/nfyjx/).

### Eligibility criteria

Types of studies eligible for review included: (1) systematic reviews, observational cohort or cross-sectional studies, retrospective electronic medical record reviews that reported prevalence and etiology of HFPS; (2) cross-sectional diagnostic accuracy studies (e.g., psychometric information on sensitivity, specificity, and positive and negative likelihood ratios) and systematic reviews that reported HFPS diagnostic criteria; (3) randomized controlled trials (RCTs), non-RCTs, quasi-experimental studies (without control group), pre vs. post intervention studies, pilot-feasibility studies, and case series/reports that reported the effect of non-pharmacological treatments.

Included participants were men and women of any age with heel fat pad pain as well as those with heel fat pad pain plus diagnoses of diabetes mellitus or rheumatoid arthritis. Exclusion criteria were cadaveric studies; animal studies; participants with heel fat pad pain plus diagnoses of acromegaly, fracture, wound, pressure sore; falanga victims; participants with a clinical diagnosis of plantar fasciopathy (defined as localized tenderness at the medial calcaneal tuberosity and morning first-step heel pain that abates after a brief period of walking); participants with unspecified plantar heel pain without differentiating between plantar fasciopathy and HFPS; biomechanical studies involving healthy participants without heel fat pad pain. We included any non-pharmacological and non-surgical interventions for HFPS, such as exercise therapy (aerobic, neuromuscular, and strength/resistance), stretching/flexibility program, movement training, manual therapy, foot orthotics, shoe insert, footwear modification/recommendation, taping, electrotherapeutics, therapeutic ultrasound, therapeutic laser, shockwave therapy, and cryotherapy. There were no restrictions on the recruitment setting (e.g., clinic, hospital, gym, school, home) in which the interventions were performed.

### Information sources and search

Using MEDLINE, Scopus, Cinahl, EMBASE, Cochrane Library, SPORTDiscus and PEDro, we conducted a comprehensive search for the prevalence, etiology, diagnostic criteria, and intervention related to HFPS on May 7^th^, 2021. The search was later updated on April 4^th^, 2022. To identify potential publications from registered trials, we searched ClinicalTrials.gov and the World Health Organization's International Clinical Trials Registry Platform (ICTRP) for pertinent registrations. Finally, abstracts, conference proceedings, and bibliographies in relevant systematic reviews (if available) were hand searched for possible inclusions. There was no restriction on the year of publication. However, only publications in English and in Human were included.

We searched individual text words in the title and abstract supplemented with Medical Subject Headings (MeSH) terms. Key terms for inclusion were’plantar fat pad’ OR’heel fat pad’ OR’plantar heel fat pad’ OR 'heel pad’ OR’plantar heel pad’ OR’fat pad atrophy’ OR’heel pad atrophy’ OR’heel contusion’ OR ‘heel bruise’ OR ‘rearfoot fat pad’ OR ‘rearfoot fat pad atrophy’ OR ‘rearfoot pad atrophy’ OR ‘rearfoot bruise’. Key terms for exclusion were ‘infrapatellar fat pad’ OR ‘patellofemoral pain’. Electronic search results were uploaded to Covidence for study selection and data extraction.

### Study selection and management

Using the Covidence platform, citations were de-duplicated; titles/abstracts were screened by two reviewers who independently determined eligibility for formal full-text reviews. Disagreements were resolved by discussion between the two reviewers or adjudication by a third reviewer. If the title/abstract did not provide sufficient information to determine eligibility, the full-text article was retrieved. Each full-text candidate article was assessed by two reviewers who independently determined the final inclusion for data extraction. Disagreements were resolved by discussion between the two reviewers or adjudication by a third reviewer.

### Data extraction and synthesis

Two reviewers independently extracted data using customized standardized data extraction forms. Extracted data from the pair of reviewers were compared for consistency. All inconsistencies were resolved by discussion between the two data extractors or adjudication by a third person. Study characteristics (e.g., design, setting, geographic location, year of data collection, publication year, and sample size) and participant characteristics (e.g., age, sex, BMI, disease duration) were summarized. Results were synthesized in three domains of (1) prevalence; (2) etiology and diagnostic criteria; and (3) non-pharmacological and non-surgical interventions. Because a scoping review aims to provide an overview by mapping the available evidence rather than a synthesized answer/result to a question, critical appraisal or risk of bias assessment is generally not recommended in scoping reviews [13, 14].

## Results

**Fig. 1:**
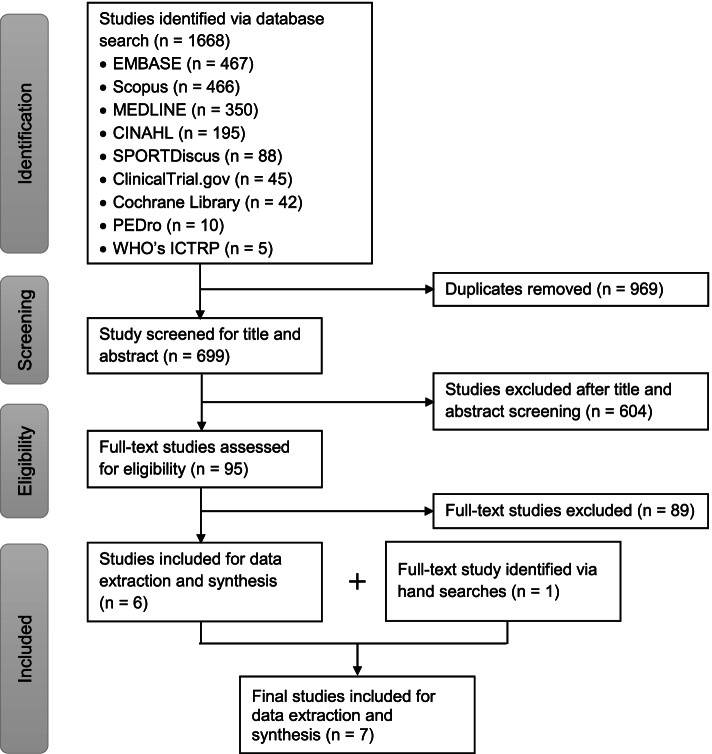
Flow chart of study selection process according to the PRISMA extension for Scoping Reviews guidelines.

**Fig. 2:**
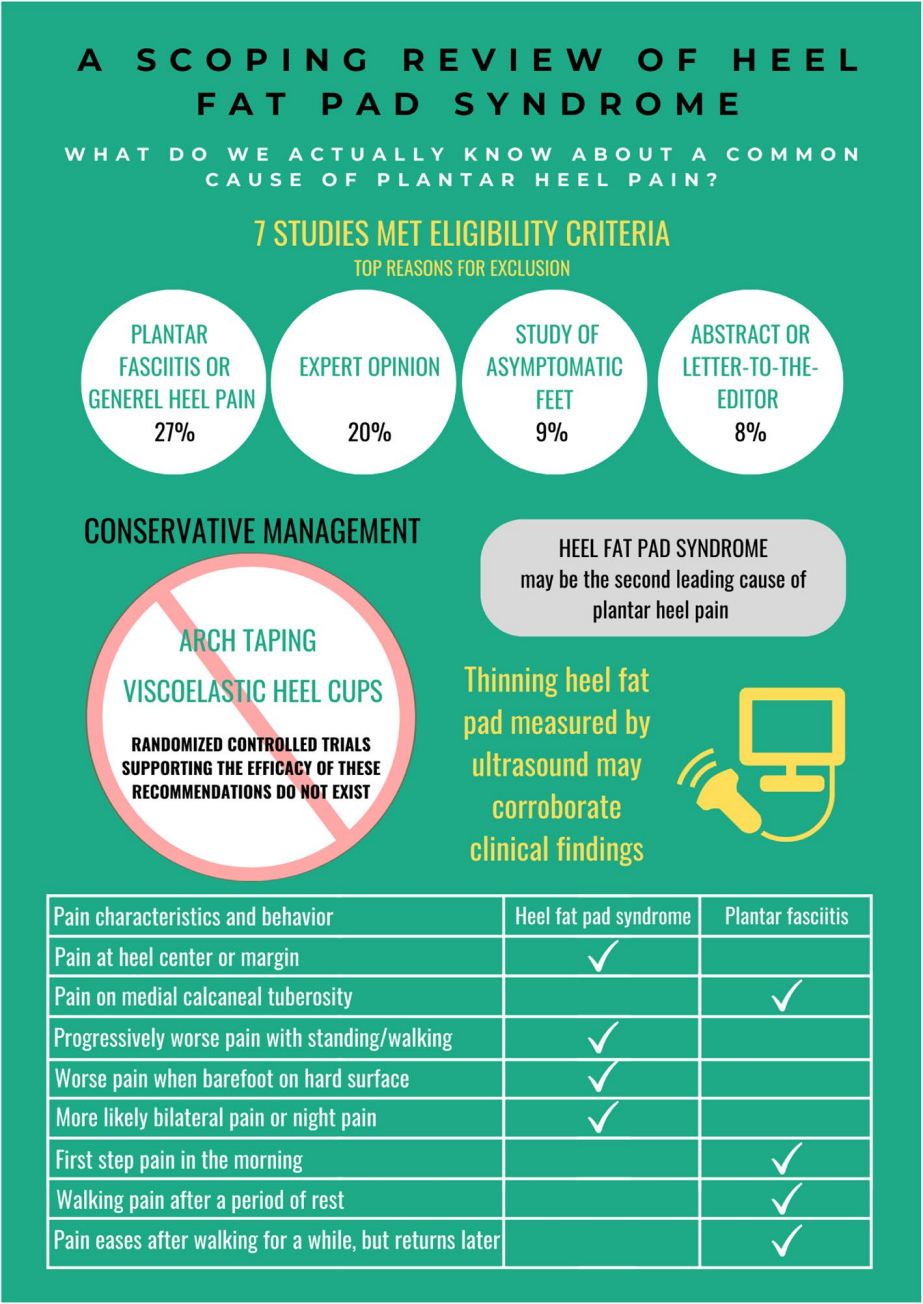
Summary findings of the scoping review

The combined database searches yielded 1668 studies. After removing duplicates, 699 studies’ titles/abstracts were screened. After screening, 95 full-text articles were reviewed for inclusion. Six studies met the inclusion criteria, and one hand-searched study was added, resulting in a final sample of 7 studies (Fig. 1). Table 1 characterized studies and study participants in the included 7 articles. Year of publication ranged from 2001 to 2021. Three articles were from Asia, 3 from Europe, and 1 from North America. Figure 2 provides a visual summary of our key findings.

**Table 1:** Study and study participants characteristics (*n* = 7)

**Study,** **Year**	**Study Design**	**Setting and Geographic Location**	**Collection Year**	**Data Domain**	**Sample Size** **(% women)**	**Age (years)**	**BMI (kg/m**^**2**^**)**	**Duration of Symptoms (months)**
Dunn et al., 2004 [4]	Population-based cohort	Community in the USA	2001–2002	Prevalence	784 (56.7%)	74.5 ± 6.0	38% had BMI ≥ 30	NA
Yi et al., 2011 [9]	Retrospective review of medical records	Foot clinic at a general hospital in South Korea	2008	Prevalence & diagnostic criteria	250 (54.4%)	43.8 ± 12.0	NR	13.3 ± 17.4
Kanatli et al., 2001 [15]	Observational case–control	NR in Turkey	NR	Etiology	106 contributing 188 feet (56.6%)	34.8 ± 12.9	26.1 ± 5.6	NR
Lopez-Lopez et al., 2019 [16]	Observational case–control	Podiatry department at a medical center in Spain	2008–2015	Etiology and diagnostic criteria	375 (47.2%)	44.7 ± 14.1	26.5 ± 5.8	NR
Balius et al., 2021 [17]	Observational case series	Outpatient clinic of sports medicine and rheumatology in Spain	NR	Etiology	9 (NR)	31 ± 8.5	NR	2.46 ± 3.72
Lin et al., 2017 [18]	Single case study	NR in Taiwan	NR	Intervention	1 male (0%)	33	NR	36 +
Chae et al., 2018 [19]	Quasi-experimental intervention (no control group)	Foot clinic at a general hospital in South Korea	2015–2016	Intervention	19 contributing 32 feet (73.7%)	51.5 ± 14.1	22.6 ± 2.6	NR

*Abbreviations:* *NA* Not applicable, *NR* Not reported

**Fig. 3:**
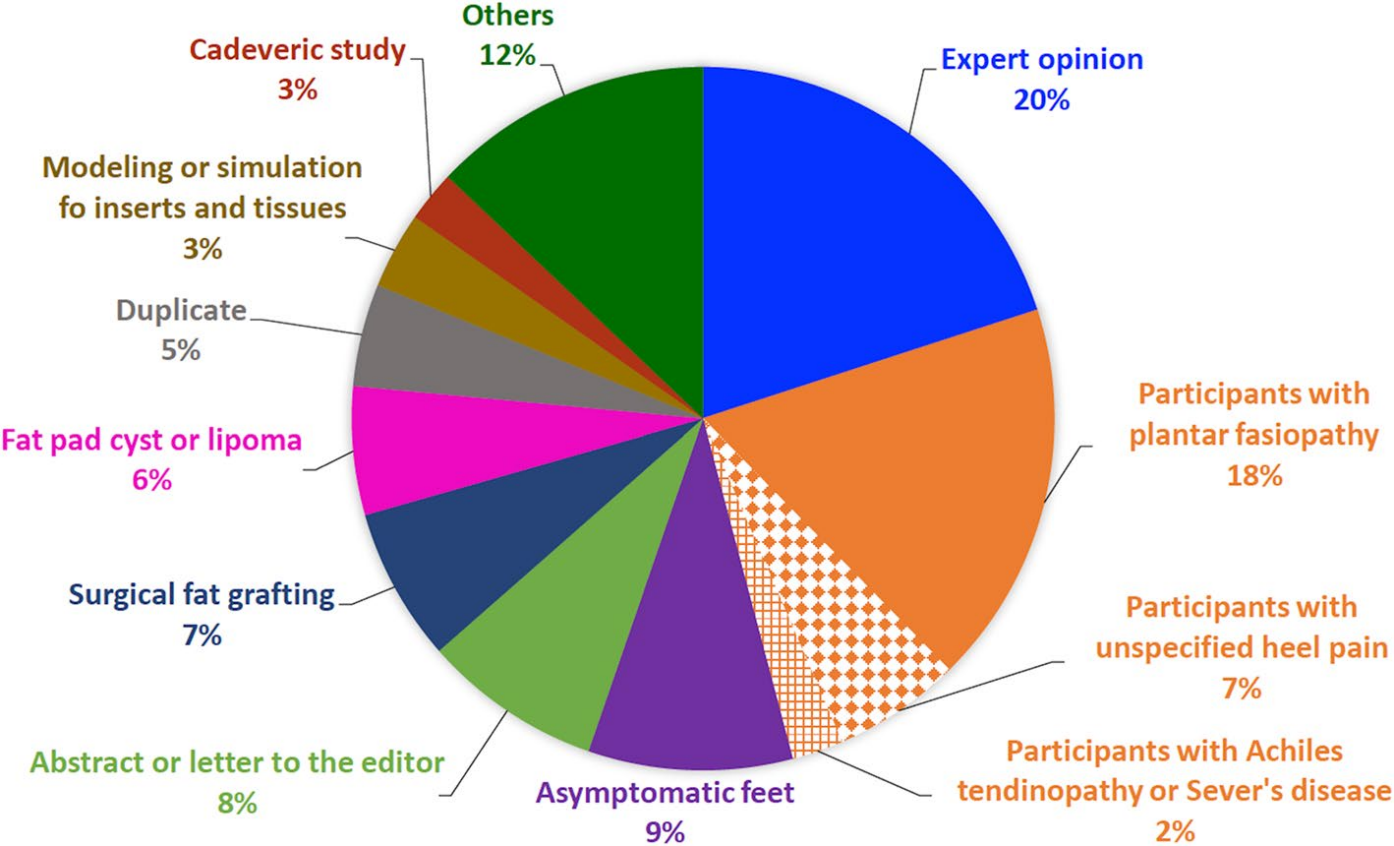
Reasons for exclusions among 89 excluded full-text articles. The top reasons were wrong patient population/diagnosis (27% in orange pies), expert opinion (20% in blue pie), others (12% in dark green pie), asymptomatic fee (9% in purple pie), and abstract or letter to the editor (8% in light green pie)

Despite the broad exploratory nature of this review, only 7 studies met our eligibility criteria. Among the 95 full-text articles reviewed, 89 were excluded. As shown in Fig. 3, the top reasons for exclusions were wrong patient population/diagnosis (*n* = 24, 27%), expert-opinion articles (*n* = 18, 20%), study of asymptomatic feet (*n* = 8, 9%), and abstract or letter-to-the-editor (*n* = 7, 8%). Notably, a lack of attention to the pathoanatomic sources of plantar heel pain was pervasive. Among 24 studies with wrong patient population/diagnoses, 16 assessed heel fat pad thickness or compressibility in patients with plantar fasciopathy; 6 included participants who had unspecified heel pain (plantar or posterior) without differentiating the source; 2 assessed heel fat pad properties in patients with Achilles tendinopathy or Sever’s disease. Of 18 expert-opinion articles, 11 were published in peer-reviewed journals and 7 were disseminated through professional magazines or online continuing education materials.

### Prevalence

Two studies reported the prevalence of HFPS [4, 9]. The Feet First Study [4] interviewed and physically examined 784 multiethnic, community-dwelling older adults, aged 65 or older, at their homes in Springfield, Massachusetts, USA in 2001–2002. Approximately 4.2% [mean standard error (MSE), 2.2%] had pain and tenderness on the heel fat pad; and 6.9% (MSE, 1.1%) had pain and tenderness on the plantar fascia. The prevalence of HFPS did not differ between sexes, but was significantly higher in Hispanic/Latino than in White or African/Black Americans.

To estimate the proportion of various pathologies among those with the general diagnosis of plantar heel pain, medical records of 250 patients with plantar heel pain at the Foot Clinic of Rehabilitation Medicine in South Korea in 2008 were retrospectively analyzed [9]. HFPS was diagnosed by the following criteria: less than 3 mm heel fat pad thickness assessed by ultrasound, pain at heel center or margin, worsening pain when barefoot or after a long period of standing. Plantar fasciopathy was diagnosed by tenderness on the medial calcaneal tuberosity and an ultrasonic hypoechoic fusiform-shaped swelling ≥ 4 mm thickness at the origin of plantar fascia. In this sample, 53% had plantar fasciopathy; 15% had HFPS, 10% had pes cavus, 9% had HFPS plus plantar faciopathy, 5% had pes planus, 4% had plantar fibromatosis, 2% had plantar fascia rupture, 2% had neuropathy or small shoe syndrome. Both studies suggested that HFPS may be the second leading cause of plantar heel pain, second to plantar fasciopathy.

### Diagnostic criteria and etiology

Four studies described diagnostic criteria or morphological/biomechanical features of HFPS [9, 15–17]. Logistic regression models identified factors associated with the diagnosis of HFPS (vs. plantar fasciopathy) in 250 patients with plantar heel pain [9]. Worsening pain during prolonged standing, pain at night, or bilateral pain greatly increased the likelihood of HFPS (odds ratios ranging from 20.9 to 25.0), while morning first-step pain and tenderness on the medial calcaneal tuberosity substantially decreased the likelihood of HFPS (odds ratios ranging from 0.04 to 0.07).

Comparing patients with (*n* = 185, age = 47.0 ± 14.7 years, BMI = 27.3 ± 6.6 kg/m^2^, 48.6% women) vs. without (*n* = 190, age = 42.0 ± 13.0 years, BMI = 25.6 ± 4.7 kg/m^2^, 45.8% women) HFPS seen in a podiatry care center, Lopez-Lopez and colleagues [16] found that ultrasound-measured unloaded heel fat pad thickness was significantly lower in those with HFPS (7.23 ± 1.39 vs. 10.36 ± 1.78 mm, *p* = 0.001, Cohen’s d = 1.959). The reduced thickness was more pronounced in women than men. Among women, the thickness discrepancy was 7.09 ± 1.44 (with HFPS) vs. 10.13 ± 1.68 mm (without HFPS). Among men, the thickness discrepancy was 7.37 ± 1.33 vs. 8.58 ± 3.43 mm. Applying the area under the Receiver Operating Curve (ROC) for identifying optimal thickness cutpoint for predicting negative HFPS, they determined a threshold of ≥ 8.77 mm for no heel pain, with a sensitivity value of 85.5% and a specificity value of 82.2%.

Another study, however, found no relationship between heel fat pad properties (heel pad thickness, compressibility, and pressure distribution) and the presence of HFPS [15]. Heel pad properties were compared between patients with HFPS (*n* = 59, age = 43.9 ± 10.5 years, BMI = 29.0 ± 5.5 kg/m^2^, 66.1% women) vs. younger healthy medical students with a lower BMI and lower proportion of women (*n* = 47, age = 23.5 ± 2.4 years, BMI = 22.5 ± 3.1 kg/m^2^, 44.7% women). Heel pad thickness in loaded and unloaded conditions were measured by radiographs. Unloaded heel pad thickness was 20.45 ± 2.89 mm (HFPS) vs. 19.55 ± 2.52 (healthy); loaded heel pad thickness was 14.02 ± 3.38 mm (HFPS) vs. 11.81 ± 2.84 (healthy). Heel pad compressibility, defined as the ratio of loaded to unloaded thickness, did not differ between groups (0.69 ± 0.14 in HFPS vs. 0.60 ± 0.11 in healthy). Peak barefoot heel plantar pressures during normal-speed walking were quantified using a pressure-recording platform embedded in the walkway. No between-group differences in peak pressure were observed (28.40 ± 6.96 N/cm^2^ vs. 31.70 ± 6.36).

In a case series of 9 patients with HFPS (age = 31 ± 8.5 years, sex and BMI unreported), ultrasound and/or MRI detected pathological heel fat pad morphologies were qualitatively described, including atrophy, fibrosis, edema, and defects in the fat pad septa with fluid in the surrounding tissues [17]. Quantitatively by ultrasound, the unloaded and loaded heel fat pad thickness was 19.8 ± 2.9 mm and 12.3 ± 2.9 mm, respectively; the compressibility index was 0.60 ± 0.09.

### Non-pharmacological and non-surgical interventions

Our systematic search only identified two interventional studies that examined the effect of non-pharmacological, non-surgical management. One was a single case report and the other was a quasi-experimental intervention. A 33-year-old man with bilateral HFPS were treated with silicone gel heel cups [18]. Right heel pad stiffness measured by shear wave elastography decreased from 65.5 kPa to 51.2 (32-day follow-up) and to 40.7 (102-day follow-up). Similar stiffness reduction was observed in the left heel pad. Objective heel pad stiffness improvement was accompanied by subjective pain relief from 10 to 3 (32-day follow-up) and to 1 (102-day follow-up) on a 0–10 visual analogue scale.

Chae and colleagues [19] examined the immediate effect of low-dye taping (LDT) and that of low-dye + figure-of-8 taping (LDT +) on heel pain and peak ambulatory hindfoot plantar pressure in 19 participants with HFPS (*n* = 32 feet, age = 51.5 ± 14.1 years, BMI = 22.6 ± 2.6 kg/m^2^, 73.7% women). It was unclear if participants were randomized into each taping group or if a cross-over design was used. No control group was included in this study design. Both types of taping significantly reduced pain [6.5 ± 1.7 (barefoot without taping), 4.2 ± 1.1 (LDT), and 3.5 ± 1.3 (LDT +) on a 0–10 visual analogue scale] and peak hindfoot pressure during walking [29.3 ± 11.9 (barefoot without taping), 26.3 ± 8.8 (LDT), 23.2 ± 7.0 (LDT +)]. There was no association between change of pain and change of peak pressure.

## Discussion

Our scoping review found a small body of original research for HFPS. This is in strong contrast to HFPS being a common cause of plantar heel pain [4, 9] and indicates a need for rigorous research to fill this knowledge gap. Only 7 studies met the inclusion/exclusion criteria with varied study designs and mixed scientific rigor. The study design of the included articles were mostly on the lower hierarchy of evidence pyramid [20, 21]: single case study (*n* = 1) [18], case series (*n* = 1) [17], case–control (*n* = 2) [15, 16], retrospective medial record review (*n* = 1) [9], quasi-experimental intervention (*n* = 1) [19]; therefore potentially introducing high risk of bias.

In this review, we excluded a significant number of expert-opinion articles in peer-reviewed or non-peer-reviewed publications. We noted that these articles sometimes support their statements by citing/interpreting other perspectives, commentaries, or non-systematic reviews, rather than empirical evidence from original research; possibly perpetuating scientifically weak knowledge. A paucity of high-quality original research for HFPS, as we have discovered in this scoping review, could have necessitated this suboptimal practice. Many excluded studies examined the heel fat pad in individuals with plantar fasciopathy. Some indiscriminately lumped HFPS and plantar fasciopathy together, notwithstanding clinical practice guidelines emphasize the importance of distinguishing one from the other [7, 11]. A number of studies were also excluded, because they assessed the biomechanical properties or morphologies of heel fat pad in asymptomatic healthy individuals; in asymptomatic patients with other chronic health conditions, such as diabetes mellitus or rheumatoid arthritis; or in cadaveric specimen.

### Prevalence

There was one prospective population-based cohort study (The Feet First Study) [4] published nearly 20 years ago. It showed that HFPS is a common foot problem in US community dwelling older adults with a 4.2% prevalence rate, second to plantar fasciopathy (6.9% prevalence rate). This finding was echoed by a retrospective record review of patients diagnosed with plantar heel pain in South Korea [9], in which 53% had plantar fasciopathy and 15% had HFPS. Given that HFPS is a distinct pathology and may be the second most frequent cause of plantar heel pain, it is unfortunate that many investigators combined participants with HFPS and plantar fasciopathy in their study sample. This oversight may have contributed to a lack of evidence-based treatment options for this condition. To our knowledge, this is the first systematic overview of research literature in HFPS. Study findings underscore the severely limited high-quality original research for HFPS. The current conservative treatment recommendations of this condition are mostly anecdotal. Future clinical trials with robust study designs are greatly needed.

### Diagnostic criteria and etiology

Damage or irritation to the heel fat pad could be caused by acute trauma (e.g., a single high-impact landing) or chronic overuse (e.g., repetitive or excessive jumping, running, or walking on hard surfaces). Fat pad atrophy are often associated with aging, rheumatological conditions, diabetes, or obesity. An accurate diagnosis of HFPS is critical for timely management. Based on Yi and colleagues’ work [9], we have summarized key differentiating pain characteristics and behaviors between HFPS and plantar fasciopathy [9] in a table of Fig. 2. Study findings [9] from the logistic regression models for identifying clinical characteristics associated with increased likelihood of HFPS or plantar fasciopathy could have been strengthened by further adjusting for potential confounders, such as age, sex, and BMI, and reporting 95% confidence interval of odds ratios.

In addition to clinical presentations, ultrasonography and magnetic resonance images (MRIs) have been used to corroborate and confirm the diagnosis of HFPS. Ultrasound quantified fat pad thickness ≤ 9 mm may be predicative of HFPS [16]. Contrarily, another study reported no thickness difference between HFPS and healthy controls [15]. An important caveat of this null finding—in this observational case–control study, the disparate age, BMI, and sex distribution between the case and control groups were unaccounted for in statistical analyses, potentially biasing study findings. The wide range (12–29 mm) of heel fat pad thickness in healthy adults [22–24] may have complicated the effort to benchmark fat pad thickness as a diagnostic criterion. According to the Physical Stress Theory [25], thicker fat pad may be present in taller/heavier person or in endurance athletes who frequently load their heel. Healthy men had thicker fat pad than women [26, 27]. BMI or sex may need to be factored into fat pad thickness interpretation of these studies. Some studies [15, 17] computed fat pad compressibility index, operationalized as the ratio of loaded to unloaded thickness, to ascertain tissue stiffness. A lack of standardized compressive force applied by the ultrasound probe to simulate loaded condition precludes across-study comparisons. Although less economical, practical, and convenient that ultrasonography, MRI could provide more nuanced morphological assessment and qualitatively characterize heel fat pad atrophy, fibrosis, edema, and septal defects [17].

### Non-pharmacological and non-surgical interventions

Viscoelastic heel cups and arch taping have been recommended for conservative management of HFPS [8, 10, 11]. Alarmingly, we could not find even a single RCT to substantiate the efficacy of these treatment strategies for HFPS. In contrast, we identified two recent systematic reviews and meta-analyses on the efficacy of foot orthoses for treating plantar fasciopathy [28, 29]. Low-dye arch taping has been shown to reduce the first-step pain in high-quality clinical trials and supported by clinical experts and patients as a reasonable first-line management for symptoms during weightbearing activities [30]. Our search yielded 2 interventional studies for HFPS [18, 19]: a single case and a quasi-experimental study. In a 33-year-old man with bilateral HFPS, heel pain decreased after 1-month and 3-month application of silicone gel heel cups [18]. Low-dye taping and low-dye plus figure-of-eight taping provided pain relief by 2 to 3 points on a 0–10 numeric pain rating scale when compared to barefoot walking [19]. In the absence of a control group, the change in self-reported pain observed in this study could be a result of regression-to-the-mean phenomenon [31] or placebo effects [32]. Our review brings to light the dire need for RCTs with scientific rigor to support evidence-based recommendations in conservative management of HFPS.

### Strengths and limitations

To our knowledge, this is the first scoping review of HFPS, comprising a comprehensive search of the prevalence, etiology and diagnostic criteria, and non-pharmacological, non-surgical management. This comprehensive and systemic scoping review closely followed the PRISMA-ScR guideline and checklist for conducting and reporting a clear, robust review. We broadly searched 9 databases and grey literatures and included all types of study designs and all years of publication. Despite our best effort and carefully defined inclusion/exclusion criteria, only 7 studies were included in the final data, which limits our ability to categorically synthesize evidence. Subpar scientific rigor in some of the included studies also dampens our confidence in part of the summarized evidence. We only included studies of people with confirmed HFPS, leaving out studies of heel fat pad biomechanics and morphologies in healthy individuals. Study participants with diagnosed plantar fasciopathy or plantar heel pain were excluded as well.

## Conclusion

The research literature for HFPS is sparse and sometimes lacking scientific rigor. In the limited evidence we have reviewed, it appears that HFPS may be the second leading cause of plantar heel pain. A number of differentiating pain characteristics and behaviors may aid in diagnosing HFPS vs. plantar fasciopathy. Thinning heel fat pad quantified by ultrasound may provide imaging corroboration of HFPS. We have identified a substantial knowledge gap for this condition, frequent inattention to distinguishing HFPS from plantar fasciopathy when describing plantar heel pain, and mostly importantly, a glaring absence of robust clinical trials examining the efficacy of commonly recommended conservative management of HFPS.

## Data Availability

All data can be made available upon request.

## Abbreviations

BMI: Body mass index
HFPS: Heel fat pad syndrome
LDT: Low-dye taping
LDT +: Low-dye taping + figure-of-8 taping
MRI: Magnetic resonance imaging
MSE: Mean standard error
RCT: Randomized controlled trial
ROC: Receiver operating curve
